# Effect of vagus nerve stimulation on blood glucose concentration in epilepsy patients – Importance of stimulation parameters

**DOI:** 10.14814/phy2.14169

**Published:** 2019-07-19

**Authors:** Harald M. Stauss, Lucienne M. Daman, Megan M. Rohlf, Rup K. Sainju

**Affiliations:** ^1^ Department of Biomedical Sciences Burrell College of Osteopathic Medicine Las Cruces New Mexico; ^2^ Department of Health and Human Physiology The University of Iowa Iowa City Iowa; ^3^ Pediatric Neurology, Department of Pediatrics The University of Iowa Iowa City Iowa; ^4^ Department of Neurology The University of Iowa Iowa City Iowa

**Keywords:** Body mass index, age, gender, stimulation on/off cycles, anticonvulsants, afferent VNS, efferent VNS

## Abstract

In previous animal experiments, we demonstrated that cervical vagus nerve stimulation (VNS) inhibits pancreatic insulin secretion, thereby raises blood glucose levels, and impairs glucose tolerance through afferent signaling. However, there are no reports suggesting that similar effects occur in patients treated with chronic cervical VNS for epilepsy. In contrast to clinical VNS used for epilepsy, where the stimulation is intermittent with cycles of on and off periods, stimulation was continuous in our previous animal experiments. Thus, we hypothesized that the timing of the stimulation on/off cycles is critical to prevent impaired glucose tolerance in epilepsy patients chronically treated with cervical VNS. We conducted a retrospective analysis of medical records from patients with epilepsy. Blood glucose levels did not differ between patients treated with pharmacotherapy only (98 ± 4 mg/dL, *n* = 16) and patients treated with VNS plus pharmacotherapy (99 ± 3 mg/dL, *n* = 24, duration of VNS 4.5 ± 0.5 years). However, a multiple linear correlation analysis of patients with VNS demonstrated that during the follow‐up period of 7.9 ± 0.7 years, blood glucose levels increased in patients with long on and short off periods, whereas blood glucose did not change or even decreased in patients that were stimulated with short on and long off periods. We conclude that chronic cervical VNS in patients with epilepsy is unlikely to induce glucose intolerance or hyperglycemia with commonly used stimulation parameters. However, stimulation on times of longer than 25 sec may bear a risk for hyperglycemia, especially if the stimulation off time is shorter than 200 sec.

## Introduction

Cervical vagus nerve stimulation (VNS) is an FDA‐approved treatment of drug‐resistant epilepsy (Nune et al., 2015) and therapy‐refractory major depression (Cristancho et al., 2011). The effectiveness of invasive cervical VNS in patients with drug‐resistant epilepsy has been demonstrated repeatedly (Pakdaman et al., 2016; Kawai et al., 2017; Vivas et al., 2017). Furthermore, some initial trials on noninvasive transcutaneous auricular VNS reported promising results (He et al., 2013; Bauer et al., 2016; Barbella et al., 2018), although not all studies demonstrated effectiveness (Song et al., 2018). Other potential clinical indications for VNS, such as heart failure (Premchand et al., 2014; Zannad et al., 2015; Gold et al., 2016), Crohn’s disease (Bonaz et al., 2016), chronic pain management (Chakravarthy et al., 2015), and obesity (Bodenlos et al., 2007; Pardo et al., 2007; Bodenlos et al., 2014) are emerging. There have also been promising reports in rats (Li et al., 2014) and humans (Huang et al., 2014; Huang et al., 2016), suggesting a potential beneficial role of noninvasive transcutaneous auricular VNS in diabetes. In contrast to these studies utilizing noninvasive transcutaneous auricular VNS, studies on the effects of invasive cervical VNS on glucose homeostasis are less promising. Our own work in rats demonstrated that acute (Meyers et al., 2016) and chronic (Stauss et al., 2018) cervical VNS impairs glucose tolerance and raises blood glucose levels by suppressing pancreatic insulin secretion through activation of afferent nerve fibers within the cervical vagus nerve (Meyers et al., 2016). In contrast, selectively stimulating efferent cervical vagus nerve fibers (by sectioning the nerve cranial to the stimulation electrode) resulted in an increase in serum insulin levels and lowered blood glucose concentrations (Meyers et al., 2016). This finding confirms earlier data from Peitl et al. (Peitl et al., 2005) who also demonstrated an increase in insulin plasma levels during electrical stimulation of the peripheral end of the cervical vagus nerve in anesthetized rats. However, selective efferent cervical VNS is currently not feasible in humans and the therapeutic effects of cervical VNS in epilepsy and depression are thought to be mediated through afferent signaling to the brain (Groves and Brown, 2005; Vonck et al., 2007; Grimonprez et al., 2015; Grimonprez et al., 2015). Stimulation of the intact cervical vagus nerve, resulting in combined afferent and efferent VNS, strongly inhibited pancreatic insulin secretion and resulted in severely impaired glucose tolerance in rats (Stauss et al., 2018). However, to our knowledge no reports exist that would indicate that chronic cervical VNS would impair glucose tolerance or increase risk for type 2 diabetes in patients with epilepsy or major depression. Thus, the primary purpose of this study was to perform a retrospective medical record analysis to test the hypothesis that chronic cervical VNS raises blood glucose levels in patients with epilepsy.

An important difference between our animal studies and VNS in a clinical setting is that a continuous stimulation protocol was used during the glucose tolerance tests in rats (Stauss et al., 2018), whereas the stimulators used in patients are programed for cycles of brief stimulation periods, followed by periods without stimulation. Very commonly used parameters for treatment of epilepsy includes alternating cycles of 30 sec on time followed by 5 min off time with no stimulation (Yamamoto, 2015). Thus, there is a possibility that the brief stimulation on time of 30 sec is not long enough to effectively suppress insulin secretion or that insulin secretion is indeed suppressed during the stimulation on time but then recovers during the subsequent 5 min without stimulation. To investigate this possibility, a secondary hypothesis of the current study was that VNS therapy in patients with epilepsy leads to stimulation‐dependent changes in blood glucose concentrations, such that the combination of a long stimulation on time with a short stimulation off time causes hyperglycemia and the combination of a short stimulation on time with a long stimulation off time has no effect on blood glucose concentration or may even reduce blood glucose concentration.

## Material and methods

### Study design and subjects

The study was approved by the Institutional Review Board at The University of Iowa and consisted of a retrospective analysis of 110 medical records from adult (age at stimulator implantation ranged from 24 to 74 years) epilepsy patients treated at the Neurology Department at the University of Iowa Hospitals and Clinics during the time period between May 2000 and August 2018. We excluded patients with a history of diabetes or patients who were taking antidiabetic drugs or systemic glucocorticoids or were pregnant. Subjects were classified into two treatment groups: patients treated with pharmacotherapy only (control group, *n* = 67) or with cervical VNS and pharmacotherapy (VNS group, *n* = 43). Nonfasted blood glucose values and body weights recorded at all clinic visits within the follow‐up period were extracted. In the VNS group, baseline values were defined as the arithmetic average of the values obtained within the time period of 10 years to 1 month prior to the stimulator implantation (one to two values per subject). In the control group, the baseline values were defined as the arithmetic average of the values reported within 1 year following the first reported value (one to five values per subject). Only values reported after the diagnosis of epilepsy were considered. For the follow‐up, all values recorded within 1 to 10 years after stimulator implantation were averaged in the VNS group (one to four values per subject). In the control group, the follow‐up values were defined as the arithmetic average of the values recorded within 4 to 14 years after the first reported value (one to six values per subject). This strategy resulted in similar total follow‐up periods in both study groups.

### Clinical variables

From the medical records we extracted date of birth, gender, height, and date of stimulator implantation (for VNS group). In addition, for each clinic visit for which a blood glucose value was reported, we also extracted date of clinic visit, blood glucose concentration, body weight, currently used medications, including antiepileptic drugs, and stimulation parameters (for VNS group).

### Nonfasted versus fasted blood glucose concentrations

The clinical records reported primarily nonfasted (random) blood glucose concentrations. Fasted blood glucose concentrations were usually available for the presurgical evaluation at the time of stimulator implantation. These fasted blood glucose values were not included in the analysis of this study. Our previous animal study indicated that cervical VNS inhibits insulin secretion in response to a glucose challenge (Stauss et al., 2018). Thus, it is expected that any potential effects of VNS on blood glucose levels would be more apparent in the nonfasted state than in the fasted state.

### Vagus nerve stimulation parameters

In all epilepsy patients of the VNS group the left cervical vagus nerve was stimulated. The stimulation current ranged from 0.25 mA to 3.50 mA (average 1.78 ± 0.17 mA), the stimulation frequency ranged from 20 Hz to 30 Hz (average 23.8 ± 1.1 Hz), and the pulse width was 250 µsec for all except two subjects, for whom the pulse widths were 130 µsec or 150 µsec, respectively. The stimulators were programed by treating physicians for repetitive cycles during which the stimulation was turned on and off automatically. We use the terms “stimulation on time” for the duration of the cycle period where the stimulation was on and we use the term “stimulation off time” for the duration of the cycle period where the stimulation was turned off. The stimulation on time ranged from 7 sec to 45 sec (average 26 ± 2 sec) and the stimulation off time ranged from 18 sec to 1200 sec (average 152 ± 58 sec).

### Effect of gender, age, body mass index, and blood glucose at baseline

To test if VNS affects nonfasted blood glucose concentration independent of gender, age, body mass index (BMI), and baseline blood glucose concentration (before stimulator implantation) a multiple linear regression analysis (Table 1) was performed, using the nonfasted blood glucose concentration at the end of the follow‐up period as the dependent parameter and the group (VNS or control), gender, age at follow‐up, BMI at follow‐up, and the nonfasted blood glucose concentration at baseline as the independent variables.

**Table 1:** Patient characteristics at baseline and at follow‐up.

Parameters	Control, *n* = 67	VNS, *n* = 43	Pooled, *n* = 110
Gender	29♀/38♂	24♀/19♂	53♀/57♂
Height (m)	1.69 ± 0.01, *n* = 67	1.68 ± 0.02, *n* = 43	1.69 ± 0.01, *n* = 110
Follow‐Up (years)	6.1 ± 0.4, *n* = 16	7.9 ± 0.7, *n* = 20	7.1 ± 0.5, *n* = 36
Duration of VNS (years)	N/A	4.5 ± 0.5, n = 24	N/A
Baseline			
Age (years)	34.7 ± 1.8, n = 67	39.6 ± 2.1, n = 39	36.5 ± 1.4, n = 106
Body Weight (kg)	81.0 ± 3.1, n = 43	79.3 ± 5.4, n = 18	80.5 ± 2.7, n = 61
BMI (kg/m^2^)	28.0 ± 1.1, n = 43	28.6 ± 1.7, n = 18	28.2 ± 0.9, n = 61
Glucose (mg/dL)	97.6 ± 2.4, n = 67	97.3 ± 3.3, n = 39	97.5 ± 1.9, n = 106
Follow‐Up			
Age (years)	38.4 ± 3.6, n = 16	44.6 ± 2.7, n = 24	42.1 ± 2.2, n = 40
Body Weight (kg)	82.4 ± 9.1, n = 10	81.4 ± 3.8, n = 20	81.7 ± 3.9, n = 30
Δ Body Weight (kg)	‐1.1 ± 6.8, n = 5	‐4.5 ± 6.2, n = 5	‐2.8 ± 4.4, n = 10
BMI (kg/m^2^)	29.9 ± 2.6, n = 10	28.4 ± 1.4, n = 20	28.9 ± 1.3, n = 30
Δ BMI (kg/m^2^)	‐0.6 ± 2.5, n = 5	‐1.4 ± 2.1, n = 5	‐1.0 ± 1.5, n = 10
Glucose (mg/dL)	98.3 ± 4.2, n = 16	99.0 ± 3.4, n = 24	98.7 ± 2.6, n = 40
Δ Glucose (mg/dL)	+5.2 ± 5.4, n = 16	+5.2 ± 5.5, n = 20	+5.2 ± 3.8, n = 36

Data were extracted from clinical files of 110 epilepsy patients. However, not all data were available from all patients, resulting in missing values. Thus, the number of subjects are provided for all parameters. BMI, body mass index; Δ, change from baseline to follow‐up. At both time points (baseline and follow‐up), there were no statistically significant differences between groups in any parameters.

### Effect of stimulation parameters

To assess a potential effect of stimulation parameters on changes in nonfasted blood glucose concentrations from baseline to follow‐up, a multiple linear regression analysis was performed using the data from subjects of the VNS group only. In the first step of the analysis, changes in nonfasted blood glucose concentrations were adjusted for potential confounding factors, including gender, nonfasted blood glucose concentration at baseline, age at stimulator implantation, duration of VNS (i.e., time period from stimulator implantation to follow‐up), and BMI at follow‐up. This adjustment was done based on the results of a multiple linear regression analysis (Table 2a) using the following equation:

$$\Delta {\text{Glu}}_{\text{Adj}}=\Delta \text{Glu}-\left({\text{Age}}_{\text{Imp}}-39.9\right)*1.17-\left({\text{Glu}}_{\text{Base}}-95.2\right)*\left(-0.69\right)$$

**Table 2:** Use of anticonvulsant drugs and benzodiazepines.

	Control, *n* = 67	VNS, *n* = 43
Anticonvulsant drugs		
Brivaracetam	ø	X
Carbamazepine	ø	X
Clobazam	ø	X
Eslicarbazepine	ø	X
Gabapentin	ø	X
Lacosamide	X	X
Lamotrigine	X	X
Levetiracetam	X	X
Oxcarbazepine	X	X
Phenytoin	X	X
Primidone	ø	X
Topiramate	ø	X
Valproate	ø	X
Zonisamide	X	X
Benzodiazepines		
Alprazolam	ø	X
Clonazepam	X	X
Diazepam	ø	X
Lorazepam	X	X

A broader range of anticonvulsant drugs and benzodiazepines was needed to control seizures in the VNS compared to the control group. ø: none of the patients in the respective group was treated with the drug; X: at least one patient in the respective group was treated with the drug.

In this equation ΔGlu_Adj_ is the adjusted change in nonfasted blood glucose concentration in mg/dL from baseline to follow‐up, ΔGlu is the actual change in nonfasted blood glucose concentration in mg/dL from baseline to follow‐up, Age_Imp_ is the age in years at the time of stimulator implantation, and Glu_Base_ is the nonfasted blood glucose concentration at baseline in mg/dL. The constants in the equation are derived from the multiple linear regression analysis shown in Table 2a. Following this adjustment for confounding parameters, a second multiple linear regression analysis was performed (Table 2b) using ΔGlu_Adj_ as the outcome parameter (dependent variable) and the stimulation parameters, including stimulation frequency, stimulation current, stimulation on time, and stimulation off time as the independent variables. The pulse width was not included in the model because all except two subjects were stimulated with a pulse width of 250 µsec.

### Power analysis

Based on the results of our animal studies (Meyers et al., 2016; Stauss et al., 2018), we hypothesized that nonfasted blood glucose levels are higher in epilepsy patients treated with the combination of pharmacotherapy plus VNS than in patients treated with pharmacotherapy only. We based our power analysis on the variability of random (nonfasted) blood glucose values measured by Bowen et al. (2018) in 7161 subjects and reported as 92.6 ± 22.4 mg/dL (mean ± SD). To detect a difference in nonfasted blood glucose levels in the order of one standard deviation in a one‐sided unpaired test at a type I (α) error of 0.05 and a power of 80% a number of 13 subjects would be required in each group. Our data set contained follow‐up glucose values in 16 control and 24 VNS patients, which allowed us to detect a difference of 0.8 standard deviations or 18.3 mg/dL at *α* = 0.05 and 80% statistical power.

### Statistical analyses

All data are presented as means ± SEM. Statistical analysis was performed using a two‐way analysis of variance (two‐way ANOVA) for one independent measure (groups) and one repeated measure (baseline vs. follow‐up). Post hoc Mann‐Whitney U tests (for comparison between groups) or Wilcoxon tests (for comparison between baseline and follow‐up values) were performed in case of significant differences in the two‐way ANOVA. For multiple linear regression analyses, linear forward and backward stepwise regression analyses using Akaike’s information criterion (AIC) were performed, using the freely available R statistical software (Chambers, 2008). Statistical significance was assumed at *P* < 0.05.

## Results

### Subject characteristics

Patient characteristics at baseline and follow‐up are provided in Table 3. At baseline, there were no statistically significant differences in age, height, body weight, BMI, and nonfasted blood glucose values between the two groups of patients. Likewise, there were no statistically significant group differences at follow‐up.

**Table 3:** Multiple linear regression analysis for the effects of the study group (VNS vs. control), gender, age, and body mass index on the nonfasted blood glucose concentration (mg/dL) at the end of the follow‐up period.

Parameter	Effect Size	Significance
Group (VNS = 1, control = 0)	N/A	n.s.
Gender (♂=1, ♀=0)	N/A	n.s.
Age (years)	+0.61	*P* < 0.05
BMI (kg/m^2^)	+0.78	*P* = 0.09
Baseline blood glucose (mg/dL)	+0.31	*P* = 0.08
Intercept	+53.56	*P* < 0.01

The multiple linear regression analysis is based on 26 subjects (10 control, 16 VNS). The effects of group and gender were not significant and were removed from the model. Model statistics: multiple *R* ^2^: 0.42; adjusted *R* ^2^: 0.34; *F*: 5.326 on 3 and 22 degrees of freedom; *P* < 0.01. N/A: not applicable; n.s.: not significant.

### Use of anticonvulsant drugs

Anticonvulsant drugs and benzodiazepines, used to control seizures in both groups of patients are summarized in Table 4. Consistent with more severe forms of epilepsy, a broader range of anticonvulsant drugs and benzodiazepines was needed for seizure control in the VNS group compared to the control group. We also noticed that a wider range of gastrointestinal drugs, including laxatives, antidiarrheal drugs, H2‐blockers, and proton‐pump inhibitors were used in the VNS group compared to the control group. However, no apparent group differences in the use of other medications were noticed.

**Table 4:** Effect of potentially confounding parameters (a) and stimulation parameters (b) on changes in nonfasted blood glucose concentrations (mg/dL) from baseline to follow‐up in patients with VNS.

(a) Identification of potentially confounding parameters			
Parameter	Mean ± SEM	Effect Size	Significance
Gender (♂ = 1, ♀ = −1)	0.05 ± 0.24	N/A	n.s.
Glucose at baseline (mg/dL)	95.2 ± 4.8	−0.69	*P* < 0.001
Age at implantation (years)	39.9 ± 2.7	+1.17	*P* < 0.001
Duration of VNS (years)	4.4 ± 0.5	N/A	n.s.
BMI at follow‐up (kg/m^2^)	28.4 ± 1.6	N/A	n.s.
Intercept	N/A	+24.44	n.s.

(b) Effect of stimulation parameters on changes in nonfasted blood glucose concentrations (mg/dL) from baseline to follow‐up, adjusted for glucose concentrations at baseline and age at implantation.			
Parameter	Mean ± SEM	Effect size	Significance
Frequency (Hz)	23.7 ± 1.1	N/A	n.s.
Current (mA)	1.9 ± 0.2	N/A	n.s.
On time (s)	25.8 ± 2.3	+0.92	*P* < 0.01
Off time (s)	96.6 ± 17.6	‐0.076	*P* = 0.06
Intercept	N/A	−11.47	*P* = 0.09

N/A, not applicable; n.s., not significant.

(a) The multiple linear regression analysis is based on 19 subjects. The effects of gender, duration of VNS, and BMI (body mass index) at follow‐up were not significant and were removed from the model. Model statistics: multiple *R* ^2^: 0.80; adjusted *R* ^2^: 0.77; *F*: 31.43 on 2 and 16 degrees of freedom; *P* < 0.001.

(b) The multiple linear regression analysis is based on 19 subjects. The effects of the stimulation frequency and stimulation current were not significant and were removed from the model. Model statistics: multiple *R* ^2^: 0.38; adjusted *R* ^2^: 0.30; *F*: 4.94 on 2 and 16 degrees of freedom; *P* < 0.05.

### Effects of cervical VNS on blood glucose

**Figure 1:**
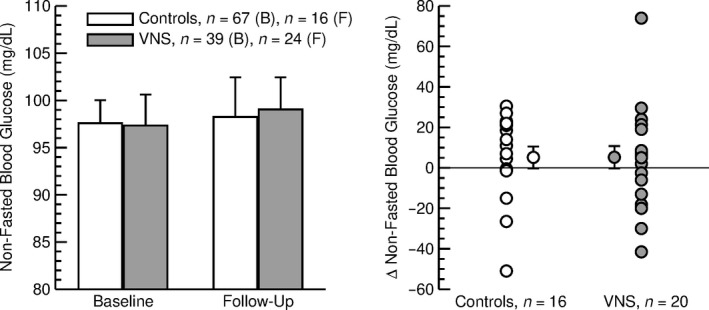
*Left:* Nonfasted blood glucose concentration at baseline (B) and at follow‐up (F) in control patients (open bars) and patients with VNS (gray bars). There were no significant differences between control patients and patients with VNS. *Right:* Changes (Δ) in nonfasted blood glucose concentration during the follow‐up period in individual control patients (open circles) and patients with VNS (gray circles). The average changes ± SEM are also shown for both groups. Only patients with baseline and follow‐up values are included. In both groups of subjects, the blood glucose concentrations at follow‐up were not significantly different from baseline values.

The data set consisted of a total number of 219 individual blood glucose values (control group: 104 baseline values from 67 patients and 31 follow‐up values from 16 patients; VNS group: 43 baseline values from 39 patients and 41 follow‐up values from 24 patients). Consistent with the exclusion of patients with diabetes or antidiabetic medications, baseline and follow‐up nonfasted blood glucose concentrations were within the normoglycemic range and were not significantly different between the two groups (Table 3 and Fig. 1, left). Specifically, at follow‐up nonfasted blood glucose levels were not significantly greater in patients with VNS (99.0 ± 3.4 mg/dL, *n* = 24) compared to the control group (98.3 ± 4.2 mg/dL, *n* = 16, Fig. 1, left). During the follow‐up period (7.1 ± 0.5 years), blood glucose increased in the overall study population (+5.2 ± 3.8 mg/dL, *n* = 36, *P* < 0.05, Table 3). However, this overall time effect was not statistically significant when considering the two groups separately (control: +5.2 ± 5.4 mg/dL, *n* = 16, *P* = 0.11; VNS: +5.2 ± 5.5, *n* = 20, *P* = 0.15; Table 3 and Fig. 1, right). Importantly, the increase in nonfasted blood glucose levels during the follow‐up period was identical in both groups.

### Effect of cervical VNS on BMI

**Figure 2:**
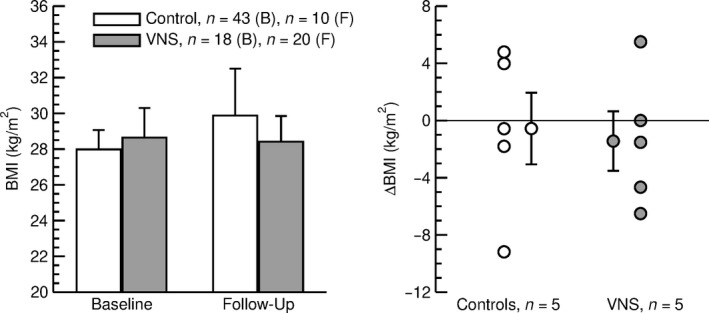
*Left:* Body mass index (BMI) at baseline (B) and at follow‐up (F) in control subjects (open bars) and patients with VNS (gray bars). At both time points, there were no significant differences between groups. *Right:* Changes in body mass index (ΔBMI) during the follow‐up period in individual control patients (white circles) and patients with VNS (gray circles). The average ΔBMI ± SEM is also shown for both groups. Only patients with baseline and follow‐up body mass index values were included. In both groups of subjects, body mass index values at follow‐up were not significantly different from baseline values.

In both groups, baseline and follow‐up BMIs were in the overweight rage (Table 3). No significant differences in BMI between the two groups were observed at baseline or follow‐up (Table 3 and Fig. 2, left). Furthermore, in both groups, no significant changes in BMI were observed during the follow‐up period (Fig. 2, right).

### Effect of gender, age, BMI, and baseline blood glucose levels

**Figure 3:**
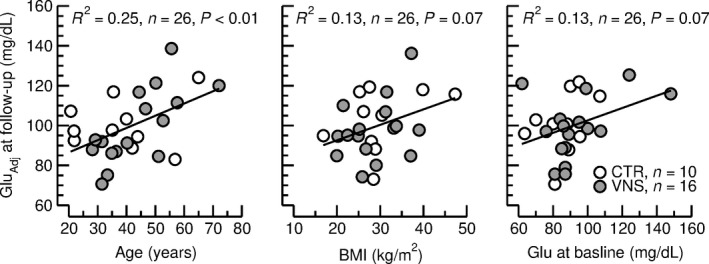
Correlations between adjusted nonfasted blood glucose concentration (Glu_Adj_) and age at follow‐up (left), body mass index (BMI, middle), and nonfasted blood glucose concentration (Glu) at baseline. In each graph, blood glucose concentrations were adjusted for the other two confounding variables according to the multiple linear regression analysis shown in Table 1. Higher age, higher BMI, and higher baseline blood glucose levels were associated with higher nonfasted blood glucose concentrations. The straight lines and the statistics (*R* ^2^, *n*, *P*) are for the linear correlations between the two parameters with the subjects from both groups pooled.

Consistent with the finding of no significant differences in blood glucose concentrations between the two groups (Table 3 and Fig. 1), the experimental group was not identified as a significant predictor for blood glucose concentration at the end of the follow‐up period in the multiple linear regression analysis (Table 1). Likewise, gender had no significant effect. As expected, higher age (*P* < 0.05), higher BMI (*P* = 0.09) and higher baseline blood glucose levels (*P* = 0.08) were associated or tended to be associated with higher blood glucose concentrations at follow‐up (Table 1 and Fig. 3).

### Effect of stimulation parameters on blood glucose concentration

**Figure 4:**
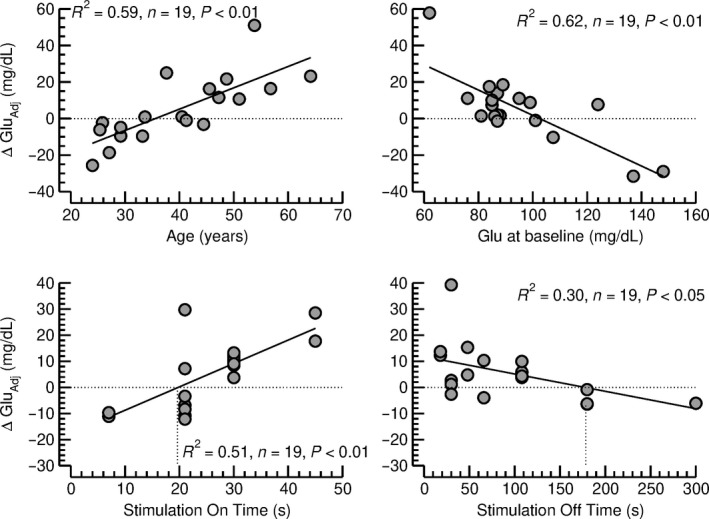
*Top row:* Effect of age at stimulator implantation (left) and nonfasted blood glucose concentration at baseline (Glu, right) on changes in nonfasted blood glucose concentration from baseline to follow‐up, adjusted for the respective other confounding factor (ΔGlu_Adj_) according to the multiple linear regression analysis shown in Table 2a. *Bottom row:* Effect of the stimulation on time (left) and stimulation off time (right) on the change in nonfasted blood glucose concentration from baseline to follow‐up, adjusted for the nonfasted blood glucose concentration at baseline and for the age at stimulator implantation (ΔGlu_Adj_). Significant linear correlations were found between both stimulation parameters and ΔGlu_Adj_. The intersections of the linear trend lines with the zero axes, were located at 20 sec for the stimulation on time and at 179 sec for the stimulation off time.

**Figure 5:**
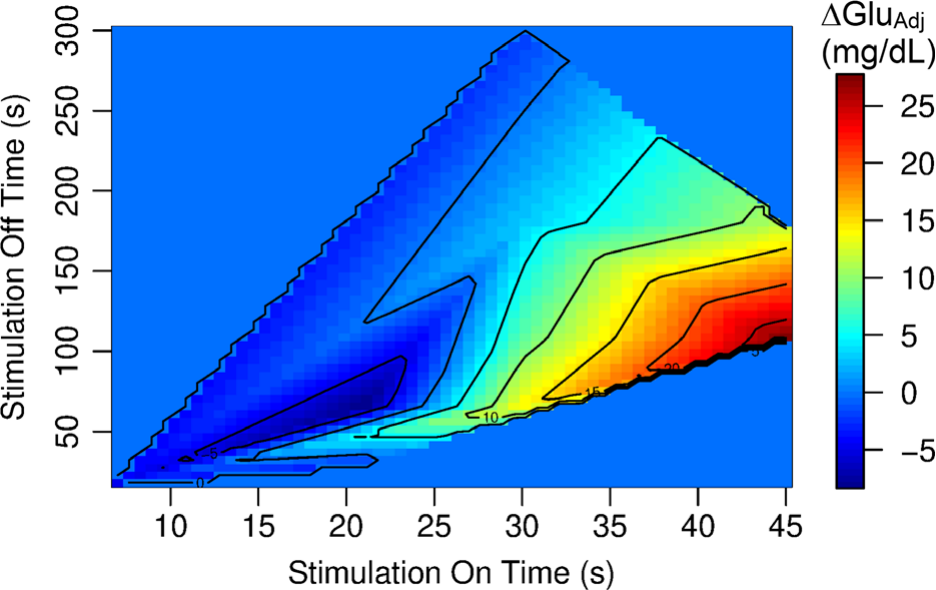
Relationship between stimulation on time (*x*‐axis), stimulation off time (*y*‐axis) and change in nonfasted blood glucose concentration from baseline to follow‐up, adjusted for the nonfasted blood glucose concentration at baseline and for the age at stimulator implantation (ΔGlu_Adj_, color coding). The color coding refers to ΔGlu_Adj_ values in mg/dL shown in the legend on the right side of the graph.

Following adjustment for the baseline glucose levels and the age of the subjects at stimulator implantation (Table 2a and Fig. 4 top), a multiple linear regression analysis identified the stimulation on time and the stimulation off time as the two stimulation parameters that correlated with the change in nonfasted blood glucose concentration from baseline to follow‐up (Table 2b). Figure 4 (bottom) illustrates that long stimulation on times and short stimulation off times are associated with increases in nonfasted blood glucose levels, whereas short stimulation on times and long stimulation off times are associated with decreases in nonfasted blood glucose levels. The neutral stimulation on and off times that were not associated with changes in blood glucose levels were 20 sec and 179 sec, respectively (intersection of trend lines with zero axes in Fig. 4, bottom). Figure 5 illustrates the relationships between stimulation on time (*x*‐axis), stimulation off time (*y*‐axis) and changes in nonfasted blood glucose levels (color coding). In this diagram, the area shaded in dark blue color (on time: 10–25 sec, off time: 50–150 sec) marks combinations of stimulation on and off times that are associated with decreases in blood glucose concentration, whereas the area shaded in yellow/orange/red hues (on time: 30–45 sec; off time: 50–200 sec) marks combinations of stimulation on and off times that are associated with increases in blood glucose levels. Interestingly, the combination of a stimulation on time of 30 sec and stimulation off time of 5 min (300 sec) that is frequently used clinically, falls in a blue shaded area and would be predicted to not increase or maybe even decrease blood glucose concentration.

## Discussion

The outcome of this study may ease concerns that emanated from our previous animal studies (Meyers et al., 2016; Stauss et al., 2018), suggesting that chronic cervical VNS may induce glucose intolerance or even diabetes in patients treated with VNS. This conclusion is based on the finding that nonfasted blood glucose concentrations were not different in patients treated with VNS (plus anticonvulsants) for 4.5 ± 0.5 years than in matched control patients treated with anticonvulsants only. In addition, changes in blood glucose concentration during the follow‐up period of approximately 7 years were not different in the two groups of patients (Table 3 and Fig. 1). Finally, the experimental group (VNS vs. control) was not identified as an independent parameter determining the follow‐up blood glucose concentration after adjusting for potential confounding factors, including gender, age, BMI, and baseline blood glucose levels (Table 1). However, it is possible that VNS increased blood glucose levels in some patients and decreased blood glucose levels in other patients depending on the timing of the stimulation on/off cycles and, therefore, no overall effect of VNS was observed. In this regard, we found that the effect of VNS on blood glucose concentration depends on the timing of the stimulation on/off cycles. Stimulation on times longer than 25 sec appears to be associated with increases in blood glucose concentrations, especially if the stimulation off time is shorter than 200 sec (Fig. 5). On the other hand, stimulation on times of less than 25 sec may reduce blood glucose concentrations, especially if the stimulation off time exceeds 50 sec. Importantly, the frequently used combination of a stimulation on time of 30 sec and a stimulation off time of 5 min (300 sec) falls within the blue area in Figure 5 that is associated with no change in blood glucose concentration.

We previously demonstrated in rats that continuous cervical VNS (without stimulation off period) inhibits pancreatic insulin secretion in the fasted state (Meyers et al., 2016) and in response to a glucose tolerance test (Stauss et al., 2018). Results of the current study demonstrate that stimulation on times longer than 20 sec (Fig. 4, bottom, left) and stimulation off times shorter than 179 sec (Fig. 4, bottom, right) are associated with an increase in blood glucose level. Thus, one may speculate that a minimum of 20 sec of VNS is required to effectively suppress pancreatic insulin secretion and to raise blood glucose levels and that a minimum of 3 min (179 sec) without stimulation is required for pancreatic insulin secretion to resume and to restore glucose homeostasis following a preceding stimulation on period that was longer than 20 sec.

Our previous animal studies also demonstrated that selective afferent VNS (stimulation of the cranial end of the sectioned vagus nerve) inhibits pancreatic insulin release, whereas selective efferent VNS (stimulation of the caudal end of the sectioned vagus nerve) stimulates pancreatic insulin release (Meyers et al., 2016). This increase in pancreatic insulin secretion in response to selective efferent VNS was also observed by others (Peitl et al., 2005). The current study suggests that short stimulation on times can actually reduce blood glucose levels (Figs. 4, bottom left). Thus, an interesting hypothesis would be that short stimulation on times (less than 20 sec) are not sufficient to effectively activate the afferent signaling pathways that ultimately inhibit pancreatic insulin secretion, but may still be sufficient to activate efferent signaling pathways that facilitate pancreatic insulin release. If this hypothesis were true, a maximal blood glucose‐lowering effect, could be achieved by combining a short stimulation on time (e.g., 20 sec) that selectively activates the efferent pathways with a stimulation off time that is long enough to prevent afferent stimulation but is also short enough to allow for a maximal number of stimulation on periods within a given time period. A stimulation off time of 75 sec seems to meet these requirements. The combination of a stimulation on time of 20 sec with a stimulation off time of 75 sec represents the timing at which the strongest decrease in blood glucose concentration was found in Figure 5 (dark blue shaded area).

We noted that there was a greater use of gastrointestinal drugs in the VNS group compared to the control group. Two possible explanations come to mind to explain this finding. First, a broader range of anticonvulsant drugs was needed for seizure control in the VNS group compared to the control group. Some of these drugs that were only used by patients in the VNS group (e.g., valproate, Table 4) are known for potential gastrointestinal adverse effects. Thus, it is possible that the greater use of gastrointestinal drugs in the VNS group is secondary to adverse effects associated with the use of anticonvulsant drugs. Second, it is possible that the greater use of gastrointestinal drugs in the VNS group reflects adverse effects of the vagus nerve stimulation. Unfortunately, the data available in the clinical records do not allow us to answer the question if the use of gastrointestinal drugs increased following stimulator implantation. However, given the dense parasympathetic innervation of the gastrointestinal system (Wood et al., 1999) we cannot rule out the possibility that the greater use of gastrointestinal drugs in the VNS group compared to the control group resulted from adverse effects of VNS.

As the study was designed as a retrospective analysis of medical records, it is not surprising that some data were unavailable for some subjects, limiting some analyses. However, the number of data points in the primary outcome parameter (i.e., blood glucose concentration) was sufficient to detect differences in blood glucose concentrations of 18 mg/dL or more with a statistical power of 80%. Given that the follow‐up blood glucose levels differed only by less than 1 mg/dL between the two groups (98.3 ± 4.2, *n* = 16 in controls vs. 99.0 ± 3.4, *n* = 24 in VNS), we are highly confident in our conclusion that cervical VNS does not put epilepsy patients at risk for impaired glucose tolerance or hyperglycemia. The use of nonfasted (random) blood glucose concentrations instead of fasted blood glucose concentrations presumably increased the variability of the blood glucose data. However, since our animal studies demonstrated that cervical VNS specifically inhibits insulin secretion in response to a glucose challenge, nonfasted blood glucose concentrations may be more sensitive to VNS than fasted blood glucose concentrations. Another limitation related to the study design is that we were not able to perform interventional experiments, such as testing if specific timings for the stimulation on/off cycles, increase (e.g., 40 sec on and 120 sec off) or decrease (e.g., 20 sec on and 75 sec off) blood glucose levels or differentially affect the responses to a glucose tolerance test. Such prospective studies may be helpful to more precisely characterize the role of VNS on glucose tolerance.

We conclude that chronic cervical VNS in patients with epilepsy is unlikely to induce glucose intolerance or hyperglycemia with commonly used stimulation parameters. Importantly, the most frequently used stimulation on/off cycle of 30 sec on and 5 min off appears to be neutral regarding blood glucose homeostasis. However, stimulation on times of longer than 25 sec may bear a risk for hyperglycemia when combined with stimulation off times shorter than 200 sec (Fig. 5). It would be interesting to further explore if short stimulation on times combined with long stimulation off times reduce blood glucose levels in patients with impaired glucose tolerance or diabetes.

## Conflict of Interest

The authors have no conflict of interest to declare.
